# MONSTER v1.1: a tool to extract and search for RNA non-branching structures

**DOI:** 10.1186/1471-2164-16-S6-S1

**Published:** 2015-06-01

**Authors:** Giulia Fiscon, Paola Paci, Giulio Iannello

**Affiliations:** 1 Institute for System Analysis and Computer Science "Antonio Ruberti" (IASI), National Research Council (CNR), Via dei Taurini 19, 00185 Rome, Italy; 2 Department of Computer, Control and Management Engineering (DIAG), Sapienza University, Via Ariosto 25, 00185 Rome, Italy; 3 Centro integrato di ricerca, Università Campus Bio-medico di Roma, Via Alvaro del Portillo 21, 00128, Rome, Italy

**Keywords:** RNA secondary structure, RNA folding prediction, Dynamic Programming Algorithm, Structural Motifs, Long Non-coding RNA

## Abstract

**Background:**

Detection of RNA structure similarities is still one of the major computational problems in the discovery of RNA functions. A case in point is the study of the new appreciated long non-coding RNAs (lncRNAs), emerging as new players involved in many cellular processes and molecular interactions. Among several mechanisms of action, some lncRNAs show specific substructures that are likely to be instrumental for their functioning. For instance, it has been reported in literature that some lncRNAs have a guiding or scaffolding role by binding chromatin-modifying protein complexes. Thus, a functionally characterized lncRNA (reference) can be used to infer the function of others that are functionally unknown (target), based on shared structural motifs.

**Methods:**

In our previous work we presented a tool, MONSTER v1.0, able to identify structural motifs shared between two full-length RNAs. Our procedure is mainly composed of two ad-hoc developed algorithms: *nbRSSP_extractor* for characterizing the folding of an RNA sequence by means of a sequence-structure descriptor (i.e., an array of non-overlapping substructures located on the RNA sequence and coded by dot-bracket notation); and *SSD_finder*, to enable an effective search engine for groups of matches (i.e., chains) common to the reference and target RNA based on a dynamic programming approach with a new score function. Here, we present an updated version of the previous one (MONSTER v1.1) accounting for the peculiar feature of lncRNAs that are not expected to have a unique fold, but appear to fluctuate among a large number of equally-stable folds. In particular, we improved our *SSD_finder* algorithm in order to take into account all the alternative equally-stable structures.

**Results:**

We present an application of MONSTER v1.1 on lincRNAs, which are a specific class of lncRNAs located in genomic regions which do not overlap protein-coding genes. In particular, we provide reliable predictions of the shared chains between HOTAIR, ANRIL and COLDAIR. The latter are lincRNAs which interact with the same protein complexes of the Polycomb group and hence they are expected to share structural motifs.

**Software availability: **the software package is provided as additional file 1 ("archive_updated.zip").

## Background

The last years have been the scene of increasing interest in long non-coding RNAs (lncRNAs), a large and heterogonous class of RNAs not translated into proteins longer than 200 nucleotides [1-4]. These novel genes appear often deregulated in cancer [5,6] and are emerging as new players of transcriptional and post-transcriptional regulation [7-9]. However, only a small subset of them has already been functionally characterized. Among them, there is a specific sub-group of lncRNAs, called *lincRNAs *(large intergenic non-coding RNAs), that reside in genomic regions which do not overlap protein-coding genes [10-14]. This positive feature in light of experimental manipulation favored them in pioneer functional studies [15]. Indeed, some lincRNAs (e.g., HOTAIR, ANRIL, and COLDAIR) constitute exemplar lncRNAs whose function can be related to their structure (see Table 1). It has been suggested as all of them interact with chromatin-remodeling complexes and specifically with the Polycomb Repressive Complex 2 [16-18]. Therefore, they can be expected to share structural motifs. Thus, a functionally characterized lncRNA (here called *reference*) can be used to infer the function of other lncRNAs that are functionally unknown (here called *target*), based on shared structural motifs. This implies to assign a structure to the reference RNA and to look for structural similarities with a target RNA.

**Table 1:** Summary of our three case-of-study lincRNAs

lincRNA	Description	Function
**HOTAIR**	HOX intergenic antisense transcript localized in the nucleus	Epigenetically silences gene expression of many loci, through the recruitment of chromatin-modifying complexes, such as PRC2, REST and CoREST. Its expression is increased in tumor cells and may have an active role in the epigenetic modulation of cancer and in mediation of the cells [11,10,39].
**COLDAIR**	Transcribed in response to biological significant events, such as a signal of cold environmental temperature	Binding the protein complex PCR2 and performing the role of a guide for the latter, determines the epigenetic repression of FLC gene (locus floral C) [40,10].
**ANRIL**	Antisense non coding transcript in the INK4 locus	Molecular scaffold for chromatin-modifying complexes PCR1 and PCR2, allows to dynamically modulating transcriptional activity [41,10]. It is implicated in a range of complex diseases including cancer and coronary heart diseases.

Although there are several tools performing the RNA secondary structure prediction, as well as detecting RNA structural similarities, they are not immediately suitable to deal with lincRNAs for two main reasons. First, large part of them are unable to efficiently treat long nucleotide sequences; second, most of the existing tools requires multiple sequence alignments, which are generally not available for lncRNAs.

In fact, an RNA secondary structure can be predicted using two main approaches: single-sequence [19] and comparative analysis [20]. The first class of methods performs prediction starting from single sequences using techniques that includes Free-Energy Minimization (e.g., Mfold [21] and RNAfold [22]) and machine learning (e.g., ContextFold [23]); while the second one makes predictions for sequence families, exploiting multiple sequence alignments to predict a consensus structure shared by all (or most) sequences in the alignment (e.g., RNAalifold [24], Dynalign [25], Carnac [26]). However, comparative analysis requires a large multi-alignment of available homologous sequences, which presently makes it not eligible approach for long and not conserved sequences of RNAs. Therefore, we focus on single-sequence methods and among them on the tools using the thermodynamic models, which result faster and do not require multiple alignments [27,28]. Thermodynamic methods rely on evaluation of the stability of a structure either quantifying free energy values [21], or assigning to each structure a probability according to the Boltzmann factor [29,30], or predicting the structure with the highest sum of base paring probability (Maximum Expected Accuracy structure [31]). Moreover, some of them can perform a global folding (e.g., Fold of RNAstructure [32] and RNAfold of Vienna RNA package), while other ones favor a local folding (i.e., *RNALfold *[33]). Since we deal with RNA of long sequences, we prefer the local folding software than the global ones. Indeed, a local folding that takes into account short-range pairs is less computational onerous and mostly more accurate than the global one [34].

For what concerns the search for structural similarities between two lncRNAs, up-to-date *Structator *[35] stands out as a computationally efficient tool to deal with long sequences. However, it does not adequately reward the rightness of the structures relative position in both the reference and the target.

In our previous work [36], we proposed a novel tool (MONSTER v1.0) that enables to detect structural motifs shared between two RNAs, taking as input only the RNA sequences independently from their nucleotides length, long as well as small. Following [35], we characterized the folding of an RNA sequence by means of a Secondary Structure Descriptor (SSD), i.e., an array of non-overlapping substructures, called *Non Branching Structures *(NBSs), and located on the RNA sequence. MONSTER v1.0 has been composed of two main core modules: (i) *nbRSSP_extractor*, to assign a unique structure to the reference RNA encoded by a set of NBSs; and (ii) *SSD_finder*, to detect structural motifs that the given target RNA has in common with the reference through an appropriate score function that takes into account the relative position among their NBSs.

On the other hand, lncRNAs as RNA molecules are not expected to have a unique fold, but appear to fluctuate among a large number of equally-stable folds [37]. Here, we present an updated version MONSTER v1.1, where we improved our *SSD_finder *algorithm in order to take into account all the alternative equally-stable structures. The improved version of *SSD_finder *includes its old version as a particular case. Hereafter, we call *SSD_finder *its improved version.

We thoroughly discuss the complexity of the *SSD_finder *and we evaluate its robustness in identifying members of some RNA families using Rfam 11.0 freely available dataset. Finally, we apply our pipeline to the three well functionally characterized abovementioned lincRNAs (i.e., HOTAIR, ANRIL, and COLDAIR).

## Methods

**Figure 1:**
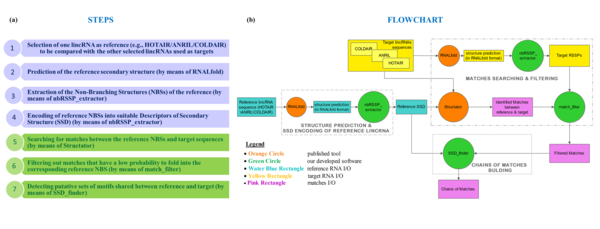
**Pipeline steps and flowcharts of the whole procedure applied to lincRNAs**. (a) The pipeline steps are explained and the two main parts of the procedure are highlighted: (1) the prediction and encoding of the lincRNA Secondary Structure Description (SSD) (blue part) and (2) the effective search engine for groups of matches (i.e., chains) common to the reference and target RNAs (green part). (b) The flowcharts of the experimental procedure is presented: given as input the reference lincRNA (HOTAIR or ANRIL or COLDAIR) and the target list composed of HOTAIR, ANRIL, and COLDAIR, the pipeline returns as output the potential structural motifs shared between reference and target. Legend: circles represent the software tools: the orange ones refer to two online available tools (i.e., RNALfold for prediction and Structator for the matches searching), the green ones refer to our developed software; rectangles represent software input and output (I/O), colored with water blue and yellow for what concerns reference and target, respectively; pink rectangles represent the final results after matching reference in the target.

**Figure 2:**
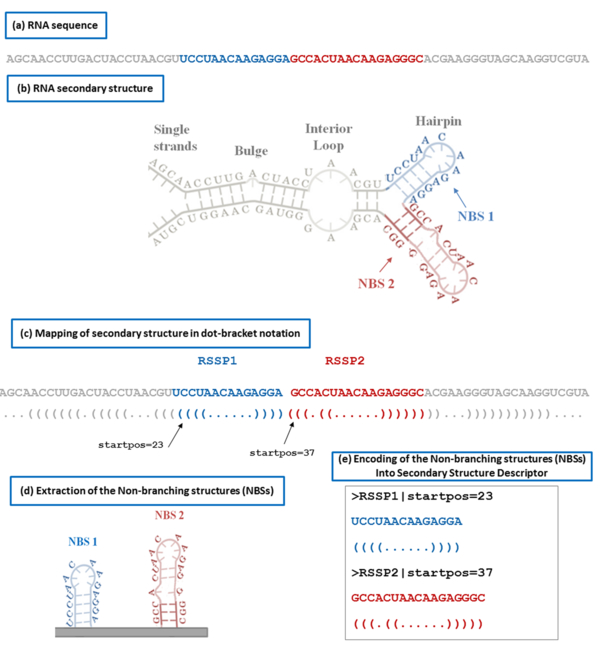
**An example of the encoding of an RNA secondary structure into a Secondary Structure Descriptor (SSD)**. (a) RNA secondary structure representation with the two highlighted Non-Branching Structures (NBSs) (red one and blue one); (b) the extraction of the two NBSs; (c) mapping of the secondary structure in the dot-bracket notation (i.e., a 3-letter alphabet where dots represent unpaired bases, open-closed brackets "()" represent the paired bases) and the visualization of the two RSSPs that are a pair of the sub-sequence and the corresponding NBS; (d) the SSD composed of the two RSSPs.

The pipeline implemented in MONSTER and sketched in Figure 1 has been applied to lincRNAs here. It can be summarized in the following steps:

1. Selection of one lincRNA as reference (e.g., HOTAIR/ANRIL/COLDAIR) to compare with the other selected lincRNAs used as targets.

2. Prediction of the reference secondary structure (using *RNALfold*).

3. Extraction of the NBSs of the reference (using *nbRSSP_extractor*).

4. Encoding of reference NBSs into suitable SSD (using *nbRSSP_extractor*).

5. Searching for matches between the reference NBSs and target sequences (using *Structator*).

6. Filtering out matches with low probability to fold into the corresponding local NBS of the reference (using our *match_filter*).

7. Detecting putative set of motifs shared between reference and target (using *SSD_finder*). *nbRSSP_extractor *makes use of the local folding tool *RNALfold *[33] from the Vienna RNA Package to obtain the folding predictions of the reference. Then, based on an *ad-hoc *selection, it extracts and encodes the more stable NBSs in a SSD (Figure 2).

*RNALfold *is a Minimum Free Energy-based predictor that returns the locally stable secondary structures of an RNA sequence according to a given parameter *L *that represents the maximum allowed distance between base-pairs. Additionally, it computes for each local structure its free energy as well as the starting position in the sequence. The output list is composed of all the possible local structures which are predicted and may overlap (i.e., more predictions correspond to an identical piece of sequence). The usage of *RNALfold *stems from its specific features: (i) it does not require homologous sequences that would be not available for lncRNAs, and (ii) it performs a local folding with lower computational costs than the other ones. In greater details, starting from a RNA sequence (Figure 2a), the predicted RNA secondary structure (Figure 2b) is broken down into separated NBSs (Figure 2d) that are conveniently represented by a dot-bracket notation (Figure 2c). Each NBS has been described by an RNA Sequence-Structure Pattern (RSSP), i.e., a pair composed of a string of bases (the sub-sequence corresponding to the NBS) and a string that represents the secondary structure in the dot-bracket notation (the NBS). In addition, a list of parameters is associated to each RSSP and composes the header line. The set of RSSPs makes up the Secondary Structure Descriptor (SSD) of the RNA sequence (Figure 2e).

*SSD_finder *takes as input each match *m_i _*(with 1 ≤ *i *≤ *n −*1, *n *number of NBSs) between the reference and target, and returns groups of matches that may correspond to common structural motifs.

We consider the chains of matches (C), as eligible chains if they:

1. consist of the largest number of consecutive NBSs;

2. exhibit similar relative distances between NBSs.

In particular, *SSD_finder *is a dynamic programming algorithm that computes for each match *m_i _*only the highest score of the best chain ending with *m_i _*using the following score function:

$$sc\left(C\right)=\overset{n}{\underset{i=1}{\sum }}P\left(m_{i}\right)+\overset{n-1}{\underset{i=1}{\sum }}Q\left(m_{i},m_{i+1}\right)$$

where:

• *P*(*m_i_*) is the weight of match *m_i _*taking into account its individual relevance (i.e., number of bases);

• *Q*(*m_i_, m_i+1_*) is a weight taking into account how much the pair (*m_i_, m_i+1_*) in the target has positions consistent with the corresponding NBSs in the reference.

Therefore, chains with the highest scores are candidates to represent structural motifs shared between reference and target RNA.

Formally, let's define:

- *R *the reference sequence;

- *T *the target sequence;

- *S *the list of NBSs extracted from the predict structure of *R*, that are sorted based on increasing starting positions;

- *s_i _*the *i*-th NBS in *S*, pos(*s_i_*) its position in *R *and len(*s_i_*) its length (1 ≤ *i *≤ *n *− 1, *n *=|*S*|);

- *M *the list of matches that are found in *T *ordered in increasing sequence positions;

- *m_i _*the *i*-th match in *M*, pos(*m_i_*) its position in *T*, len(*m_i_*) its length, and nbs(*m_i_*) the NBS in *S *which *m_i _*corresponds to;

- ind(·) an operator that starting from 1 returns the index of the argument (i.e., either a NBS in the list *S*, or a match in the list *M*);

Specifically, matches of a list *M *are pairs consisting of:

- index *i *of *S *of the matching NBS *s_i_*;

- position pos(*m_i_*) of matching subsequence of *T *(i.e., a subsequence that can fold into *s_i _*based on the base pairing rules).

Note that *M *is composed of all potential matches, including overlapping matches. Moreover, for what concerns the list *S *of NBSs we have to distinguished two possible cases:

• *case S*^(1)^: the list *S *is composed of only non-overlapped NBSs that form a unique prediction of the *R *structure. Formally, *S *contains only *s_i _*such that the pos(*s_i_*)+ len(*s_i_*) ≤ pos(*s*_*i*+1_) condition is satisfied for any *i*;

• *case S*^(k)^: the list *S *includes even overlapped NBSs that form *k *possible alternative predictions of *R *structure. Formally, *S *contains *s_i _*such that may exist indices *i, j *with *i*<*j *implying pos(*s_i_*)+ len(*s_i_*) > pos(*s*_*i*+1_).

Note that *case *S^(1) ^is the only option implemented in [36]. Furthermore, we remark that our *nbRSSP_extractor *can be switched between the two specific options to enable extracting either only the non-overlapped NBS from the *RNALfold *predictions (in the *case S*^(1)^) or all the even overlapped NBSs (in the *case S*^(k)^), according to the analyzed case study.

Finally, we define a *chain $C=\left\{m_{j_{1}},m_{j_{2}},\cdots \;,m_{j_{n}}\right\}$*of matches in *M *as a group of matches satisfying the following conditions ∀i,1≤i≤n-1 with *n=*|M|:

(i) ind (nbs($m_{j_{i}}$)) < ind (nbs($m_{j_{i+1}}$))

(ii) pos($m_{j_{i}}$)+length($m_{j_{i}}$) ≤ pos ($m_{j_{i+1}}$)

In the following, we will denote the matches in a chain as *m*_1_,..., *m_n _*since the condition (ii) implies that *C *is sorted according to increasing positions in *T*, which implies that $j_{i}<j_{i+1},\forall_{i},1\leq i\leq n-1$, and hence there are no ambiguities on match indices.

More details about the whole procedure are explained in specific sections of the additional file 2 (*Supplementary_Material*), in the additional file 3 (*User_Guide*), and additional file 4 (Figure S1).

### SSD_finder complexity

*SSD_finder *algorithm looks for the most meaningful chains of matches using the dynamic programming and regarding for all the matches *m_i _*∈ *M *only the chains ending with *m_i _*that have the highest score. Let now consider *f*=ind(*m*_*i+*1_) and *g*=ind(*m_i_*) such that the condition (i) and (ii) are satisfied.

We use a score function *Q*(*f, g*) that assigns a score to the pair of matches (*m_i_, m_i+1_*). The complexity of our *SSD_finder *can be evaluated by counting the times the *Q *function is called. The dynamic programming algorithm calls the *Q *function once for any pair (*f, g*) with *g*<*f*. Hence, the cost of the programming algorithm is:

$$\overset{n}{\underset{f=1}{\sum }}\overset{f-1}{\underset{g=1}{\sum }}h\left(f,g\right)$$

where *h*(*f, g*) is the cost of the call to function *Q*(*f, g*).

In the case *S*^(1)^, *h*(*f, g*) is a constant for any *f, g*, leading to a total cost equal to $\frac{n\left(n-1\right)}{2}$. Therefore, the computational complexity of the *SSD_finder *algorithm when we have only a unique prediction of *R *is equal to *O*(*n*^2^).

In the case *S*^(k)^, *h*(*f, g*) accounts for the alternative perditions of the same structure of *R*. Firstly, we remark that *Q*(*f, g*) weight *S *how much the pair (*f, g*) has positions consistent with the corresponding NBSs in *R*. Therefore, the *Q *function depends on a distance *d*(*f, g*) between *f *and *g *with *g<f*. We define the distance *d*(*f, g*) as 1 plus the maximum number of non-overlapped NBSs *s_k _*such that *g<k<f*. Note that according to this definition, if there are no non-overlapped NBSs between *s_f _*and *s_g_, d*(*f, g*) = 1.

To compute *d*(*f, g*), the following algorithm can be used:

1   Initialize l=f; D={ };

2   Compute

    $S=\left\{k|g<k<f\wedge pos\left(s_{k}\right)\leq pos\left(s_{l}\right)\wedge pos\left(s_{g}\right)+len\left(s_{g}\right)\leq pos\left(s_{k}\right)\right\}$

3   If S≠{ } compute

          j=max(S)

D=D∪j

          *l *= *j *; and repeat step 2,

   otherwise,

          d(f, g)=1+|D|; and stop.

Since the algorithm must consider all NBSs between *f *and *g*, its complexity is (*f*-*g*), and this is also the cost of function *Q*(*f, g*). Hence, the complexity of dynamic programming algorithm is:

$$\overset{n}{\underset{f=1}{\sum }}\overset{f-1}{\underset{g=1}{\sum }}f-g=\overset{n}{\underset{f=1}{\sum }}f\left(f-1\right)-\frac{f\left(f-1\right)}{2}=\frac{1}{2}\overset{n}{\underset{f=1}{\sum }}\left(f^{2}-f\right)$$

that results equal to $\frac{n\left(n^{2}-1\right)}{6}≅O\left(n^{3}\right)$ according to the summation formulae of $\overset{n}{\underset{f=1}{\sum }}f^{2}=\frac{n\left(n+1\right)\left(2n+1\right)}{6}$ and $\overset{n}{\underset{f=1}{\sum }}f=\frac{n\left(n+1\right)}{2}$. Therefore, the computational complexity of the *SSD_finder *algorithm when we have more alternative predictions of *R *is equal to *O*(*n*^3^).

### Validation procedure

In the following, we define the experiments carried out to perform the *SSD_finder *validation.

The validation procedure aims to test the results of our algorithm to identify members of RNA families obtained from the Rfam 11.0 database [38]. It can be summarized in the following steps: (i) we build up a target dataset composed of several Rfam families (i.e., more than 700 sequences belonging to different families); (ii) we select a whole sequence belonging to a given Rfam family as reference; (iii) we run the *SSD_finder *obtaining the score of each sequence; (iv) we sort all the sequences of the target dataset in a decreasing order according to the returned score.

In greater details, among the reference Rfam families, we identify the following ones: (i) the Citrus tristeza virus replication signal family (RFAM Acc.:RF00193) composed of 44 sequences with an average length of 267 nucleotides (nt) (longest sequence length = 274 nt, shortest sequence length = 214 nt); (ii) the small ncRNAs OxyS family (RFAM Acc.: RF00035) composed of 299 sequences with an average length of 109 nt (longest sequence length = 114 nt, shortest sequence length = 68 nt); (iii) the lncRNAs family HAR1A (RFAM Acc.: RF00635) composed of 66 sequences with an average length of 118 nt (longest sequence length = 135 nt, shortest sequence length = 96 nt). Furthermore, the target dataset is composed of 723 sequences, including the abovementioned families and a subset of families randomly extracted from the Rfam 11.0 (http://rfam.sanger.ac.uk/) and RNAstrand v2.0 (http://www.rnasoft.ca/strand/) databases, whose shortest, longest, and average sequence length are equal to 43 nt, 551 nt, and 201 ± 8 nt, respectively.

## Results and discussion

### Validation

To evaluate the robustness of *SSD_finder*, we extensively test its performances in the identification of members of an RNA family. Specifically, the latter has been obtained from the online freely available Rfam 11.0 database [38], which consists of a curated collection of related RNAs. Note that the Rfam database selects only a short portion of the sequences which belongs to the Rfam families.

In the following, we explain the results of the experiments that we carried out to perform the *SSD_finder *validation.

With reference to our validation procedure explained in the last subsection of *Materials and Methods*, in [36] we focused on the recognition of the consensus structure of some Rfam families (i.e., RF00193, RF00035, RF00635) for the selected short portion of these sequences. We observed that our *SSD_finder *was able to detect more than 80% of the members of families with high specificity. In Figure S2, additional file 5, we report the trend of the score computed by *SSD_finder *with respect to the target sequences to be covered.

Here, we add noise in the input of *SSD_finder*, considering the whole original sequence for the member of the investigated family. In greater details, we test the *SSD_finder *performances in the identification of a whole sequence randomly chosen from those belonging to the Rfam families. In fact, the Rfam families are composed of manually-curated fragments of RNA sequences. However, we aim to identify the whole sequence (reference RNA) as belonging to the right Rfam family among several fragments of sequences (target RNA). In this way, it should be more difficult to pick the right family to which the sequence belongs. However, we find that *SSD_finder *ranks the reference sequence in the top three of the score ordered list. Thus, our *SSD_finder *succeeds in finding the right family to which the whole sequence belongs even if the target dataset is only composed of sequence segments that made the detection harder.

More details about the score ranking and all the validation procedure are discussed in a specific section of the additional file 2 (*Supplementary_Material*) and in the additional file 6 (Figure S3).

We conclude that our algorithm is highly effective to discriminate the sequence belonging to the reference Rfam family.

### Prediction

We study three lincRNAs, HOTAIR, ANRIL, and COLDAIR that constitute exemplar lincRNAs whose function seems to be related to their structure (Table 1).

We apply both the *SSD_finder *flags, above referred as *case S*^(1) ^and *case S*^(k)^, for each combination of the three lincRNAs. The results are reported in Table 2 and Table 3, respectively. In greater details, in both Tables the score values computed by equation (1) and the length of the best chain of matches between reference and target lincRNA have been reported for each lincRNA.

**Table 2:** Results of chain predictions about the HOTAIR, ANRIL and COLDAIR lincRNAs when a unique prediction for the reference lincRNA is considered (case S^(1)^).

Target	HOTAIR		ANRIL		COLDAIR	
**Reference (number of RSSPs)**	***Score***	***Chain length***	***Score***	***Chain length***	***Score***	***Chain length***

HOTAIR (67 RSSPs)	**224.5**	67	7.24	5	6.80	5
ANRIL (96 RSSPs)	4.78	4	**351.7**	96	5.98	4
COLDAIR (25 RSSPs)	3.06	2	5.04	3	**95.9**	25

**Table 3:** Results of chain predictions about the HOTAIR, ANRIL and COLDAIR lincRNAs when alternative predictions for reference lincRNA are considered (case *S*^(k)^).

Target	HOTAIR		ANRIL		COLDAIR	
**Reference (number of RSSPs)**	***Score***	***Chain length***	***Score***	***Chain length***	***Score***	***Chain length***

HOTAIR (241 RSSPs)	**271.9**	74	38.5	21	18.6	10
ANRIL (335 RSSPs)	29.4	18	**412.4**	111	18.1	10
COLDAIR (98 RSSPs)	13.9	7	13.8	7	**111.8**	25

In Table 2 we show performance of *SSD_finder *in *case S*^(1)^, where only a unique prediction of the reference structure has been considered. This corresponds to the only case implemented in MONSTER v1.0. When each lincRNA is run against itself, the chain length found in the target is exactly equal to the number of RSSPs of the reference. To such a specific match MONSTER v1.1 assigns the highest score (bold numbers). We conclude that *SSD_finder *succeeds to dredge the whole SSD of the reference lincRNA in the target lincRNAs list.

However, when each lincRNA is run against the others, *SSD_finder *works quite well. For example, HOTAIR (reference) versus ANRIL (target) show a shared chain of length 5 and score 7.24. It means that 5 non-branching structures are common between HOTAIR and ANRIL, building a common structural motif. Following the idea that a way of functioning of lincRNAs relies on their structures, this result could be a functional signature to infer a putative mechanism of action shared by HOTAIR and ANRIL.

In Table 3 we report the performances of *SSD_finder *on the lincRNA in *case S*^(k)^, or when it takes into account all alternative structure predictions of the reference lincRNA. This can be clearly seen by the number of reference lincRNA RSSPs that is drastically higher than that of the corresponding field of Table 2. Indeed, the SSD of HOTAIR in Table 2 is composed of 67 non-overlapped RSSPs that constitute a unique prediction of its structure, while the corresponding SSD in Table 3 is composed of 241 RSSPs, including the overlapping ones that represent the alternative predictions. Even here, *SSD_finder *succeeds to identify the entire chain of lincRNA reference, providing different possible configurations of such a chain. In fact, since in this case even alternative NBSs of the reference lincRNA are taken into account, the chain lengths are always greater than the corresponding ones in Table 2 whose NBSs did not overlap. Furthermore, it found some chains of matches shared between different lincRNA (e.g., HOTAIR versus ANRIL show a common chain of 21 RSSPs and 38.5 score) that can suggest as abovementioned some putative common structural motifs.

Interestingly, looking at the list of common RSSPs between all the pairwise comparisons (e.g., HOTAIR and ANRIL; HOTAIR and COLDAIR; etc...), we have noticed the following meaningful characteristics (data not shown):

(i) 2 RSSPs of the chain shared between HOTAIR and ANRIL are also common between HOTAIR and COLDAIR, (ii) 4 RSSPs of the shared chain between ANRIL and HOTAIR are also among those ones that compose the shared chain between ANRIL and COLDAIR, and finally (iii) 3 RSSPs of the 7 ones that compose the shared chain between COLDAIR and HOTAIR are even among those ones of the COLDAIR and ANRIL shared chain. Therefore, we conclude that some structural motifs are shared among all the three lincRNAs, pointing to putative shared function of HOTAIR, ANRIL and COLDAIR.

Note that both Tables are not symmetrical for the exchange of the reference with target and *viceversa *and the results differ depending on which one is chosen as reference. This is due to the representation of the secondary structure to be searched for in the target sequence that is the result of a structure prediction and hence leads to some false positive and false negative values. Of course, the group of RSSPs that can be putative common structural motifs should be those ones found with a reference.

Finally, in order to give a statistically significance to our predictions, we computed 1000 shuffled versions of the three analyzed lincRNAs and we compared the score of the original comparisons with respect to those obtained by unrelated sequences. In this way, we are able to assign p-values to the scores of the original comparisons. In particular, we computed the zscore distribution

($zscore=\frac{\text{x}-\mu }{\sigma }$*with *μ = *mean*, σ = *standard deviation*) of the highest scores of the unrelated sequences. Then, we located the original scores on this distribution and we found that they rely on the tails of the distribution corresponding to a p-value. The supplementary Figure S4, additional file 7, sketches the computed zscore distributions and the zscore corresponding to our original score. We noticed that our original scores rely on the tail of the distribution corresponding to a p-value ≈ 0.

## Conclusions

We applied MONSTER v1.1 to search for the potentially shared structural motifs of three lincRNAs: HOTAIR, ANRIL, and COLDAIR. We found that *SSD_finder *has been resulted highly specific in recognizing the lincRNA selected as reference in the target list. Moreover, it predicted putative shared chains of matches between different lincRNAs that can be used to point to a common way of functioning. Finally, our method can aid to assign a putative function to an uncharacterized RNA relied on their secondary structures.

## List of abbreviations

• lncRNA = long non-coding RNA;

• lincRNA = large intergenic non-coding RNA;

• NBS = Non-Branching Structure;

• RSSP = RNA Sequence Structure Pattern;

• SSD = Secondary Structure Descriptor.

## Software availability

The developed software package is available as supplementary file (zipped file named: "archive_updated.zip", additional file 1). Please refer even to the archive.zip provided as supplementary material of our work [36] to obtain the previous version of our procedure^.^

## Competing interests

The authors declare that they have no competing interests.

## Authors' contributions

GI and PP conceived the core idea of the research. GI and GF developed and implemented algorithms. GF tested extensively the implementation, collected and analyzed experimental data. GF and GI made interpretation of data. All the authors wrote the paper. All the authors read and approved the final manuscript.

## Supplementary Material

Additional file 1 **It contains the MONSTER_v1**.1 software package with the updated version of SSD_finder, the file "INSTALL" that guides the package installation, the "data" and "example_data" folders which store all the needed files, including example input file and a script to run the whole procedure. Click here for file

Additional file 2 **It contains details about the MONSTER procedure, the validation procedure, and the prediction results**. Click here for file

Additional file 3 **It contains a detailed guide for MONSTER setting up and additional advanced information about the package algorithms and their application**. Click here for file

Additional file 4 **Figure S1 - Relevance of the Q term of score function (1)**. It contains the supplementary figure that depicts an example of the Relevance of the Q term of the score function into chaining. An example of the chaining step (step 7) that shows the relevance of evaluating the distance among the RSSPs along with the number of RSSPs to select the best chain of matches. Click here for file

Additional file 5 **Figure S2 - Chain scores evaluated from SSD_finder**. It contains the supplementary figure that depicts the chain scores evaluated from SSD_finder. Each panel represents the efficiency of our SSD_finder in the classification of the members of the four analyzed Rfam families (i.e., (a) RF00193, (b) RF00035, (c) RF00635, and (d) RF1975). The y axes represent the score of our algorithm, evaluated as in equation (1) in our manuscript; the × axis represents the number of RNA sequences that constitute the database used as target in the chaining validation. This database includes the four selected families and a subset of families randomly extracted from the Rfam and RNAstrand databases (more than 700 sequences in total). In each case, the score computed by SSD_finder drastically decreases approaching to the number of sequences that corresponds to the number of the family members, allowing a clear identification of the exact number of detected members. Click here for file

Additional file 6 **Figure S3 - Ranking results of the chain score of SSD_finder in the validation procedure**. It contains the supplementary figure that depicts the rank results. Each panel represents the efficiency of our SSD_finder in identifying the right Rfam family in at least the three positions. (a) Table with the ranking results, whose first column represents the sequences used as target (1=fragment of sequence belonging to the right detected Rfam family; 0=other sequences that do not belong to the detected Rfam family). (b), (c) Bar plots that represent the graphical representation of the ranking results. The y axes represent the normalized score of our algorithm, evaluated as in equation (1) in our manuscript; the × axes represent the number of RNA sequences that constitute the database used as target in the chaining validation. This database includes the Rfam family to which the whole sequence used as reference belongs and a subset of families randomly extracted from the Rfam database (more than 700 sequences in total). The first group of plots (b) sketches the first seven ranking results, the second one (c) represents the whole set of sequences. Click here for file

Additional file 7 **Figure S4 - Distribution of the zscores**. It contains the supplementary figure that depicts the zscore distributions. Each panel represents the score distribution of zscore using a shuffled version of the lincRNA joint with the score of our original comparison: (a) HOTAIR has been chosen as reference and the shuffled versions of ANRIL as target; (b) HOTAIR has been chosen as reference and the shuffled versions of COLDAIR as the target; (c) COLDAIR has been chosen as reference and the shuffled versions of ANRIL as target. It has been noticed how in all the cases our score is statistically significant with respect to the random distribution, yielding on the tails of distribution and corresponding to a p-value ≈0. Click here for file

## Declarations

Publication of this article has been funded by the Institute for Systems Analysis and Computer Science "A. Ruberti" (IASI) of the italian National Research Council (CNR) and by The Epigenomics Flagship Project (Progetto Bandiera Epigenomica) EPIGEN funded by Italian Ministry of Education, University and Research (MIUR).

This article has been published as part of *BMC Genomics *Volume 16 Supplement 6, 2015: Proceedings of the Italian Society of Bioinformatics (BITS): Annual Meeting 2014: Genomics. The full contents of the supplement are available online at http://www.biomedcentral.com/bmcgenomics/supplements/16/S6.
