# Supplementary material for: MONSTER v1.1: a tool to extract and search for RNA non-branching structures
Source: BMC Genomics. 2015 Jun 1;16(Suppl 6):S1. doi: 10.1186/1471-2164-16-S6-S1 (PMC4460781; doi:10.1186/1471-2164-16-S6-S1)
Supplement: Additional file 2 — It contains details about the MONSTER procedure, the validation procedure, and the prediction results. [file 1471-2164-16-S6-S1-S2.pdf]

Supplementary Material

MONSTER v1.1: a tool to extract and search for RNA non-branching structures

Giulia Fiscon, Paola Paci, and Giulio Iannello

This documents includes more additional details about the MONSTER procedure, the validation procedure, and our prediction results.

1 Extraction of the reference RNA non-Branching structures

In this section, we provided more details about the step 3 and step 4 of our procedure performed by the core module *nbRSSP_extractor*.
First of all, we need secondary structure predictions of the reference RNA. To this aim, we make use of RNALfold tool that provides locally stable sub-structures according to a given parameter L, representing the maximum allowed distance between a base-pair. RNALfold also computes for each sub-structure both the starting position in the sequence and its free energy. It is worth noting that two or more sub-structures may overlap (i.e., more predictions correspond to an identical piece of sequence). Thus, RNALfold gives as output a list of all possible local sub-structures. If we are interested in a unique prediction of reference RNA, it has to be composed by non-overlapping sub-structures. The core module nbRSSP_extractor extracts a set of non-branching structures that do not overlap, representing the structure of the reference RNA. We introduce the following definitions:

Definition 1
Two local predictions of non-branching structures are the same sub-structures if: (i) their structural description coincides in length, base-pairs, and unpaired bases, and (ii) they are placed at the same initial position on the RNA sequence.

Definition 2
Two local predictions of non-branching structures that are not the same sub-structures have a non-branching sub-structure in common if: (i) the length of their external loop is the same, and (ii) this loop is placed at the same positions on the RNA sequence.

Note that the common sub-structures of *Definition 2* between two local predictions of non-branching structures extend before and after the external loop as long as paired and unpaired bases of the two predictions coincide. Therefore in the following, unless differently stated, the common part is the larger possible. The module nbRSSP_extractor extracts from the $i-th$ sub-structure ($i = 1 \dots N$ with N number of the sub-structures provided by RNALfold) the set of non-branching structures (nbs_i). Let $u^{(i)}$ be one of the non-branching structures belonging to nbs_i of the $i-th$ sub-structure and $v^{(j)}$ one of the non-branching structures belonging to nbs_j of the $j-th$ sub-structure. It may happen that $u^{(i)}$ and $v^{(j)}$ coincide or have a non-branching sub-structure in common (i.e., either $u^{(i)}$ is strictly contained in $v^{(j)}$, or $v^{(j)}$ strictly contains $u^{(i)}$, or the common part is strictly contained in both $u^{(i)}$ and $v^{(j)}$). Based on this observation, nbRSSP_extractor constructs the set of all different Non-Branching Predictions (NBP), including the ones that are in common between

any pair of different predicted local structures computed by RNALfold.For each $k \in NBP$, the module nbRSSP_extractor computes the mean free energy per base defined as:

$$me_{pb}(k) = \frac{1}{n(k)} \cdot \sum_{i=1}^{n}(k)\frac{e_i}{l_i} \tag{1}$$

where e_i is the free energy of the $i-th$ sub-structure provided by RNALfold, l_i is its length, and $n(k)$ represents the occurrences of k in the structure predictions.Then, nbRSSP_extractor sorts NBP according to decreasing me_{pb} and, starting from the first element, constructs a list of NBSs by selecting all predictions that do not overlap. This list is then reordered according to increasing position in the sequence.
For what concerns the alternative (i.e., more than one) representations of the reference RNA structure, starting from the RNALfold prediction our nbRSSP_extractor algorithm extracts the set of NBSs that can be even overlapped, where overlapped NBSs belong to different alternative structures of the same reference sequence.

2 Filtering out of low-probability matches

In this section, we provided more details about the step 6 of our procedure performed by the MONSTER module *matches_filter*. It filters out unlikely matches obtained from the step 5 of our procedure (i.e., match searching using Structator).
We defined "*unlikely* match" a target subsequence that matches with a RSSP of the reference, but can not fold in such a RSSP according to the structure prediction tools. In fact, the Structator module *afsearch* looks for matches taking into account the potential base-pairing as the only constraint. This represents only a necessary condition and it could produce many false positives. To discard unlikely matches, we apply again the equal schema used to predict the structural motifs of the reference sequence (step 3-4), with the difference that we use a less selective criterion to accept sub-structures of putative matches: only the matches whose NBSs appear in the list predicted by RNALfold/nbRSSP_extractor analysis are retained. Since the contribution of a match to find not trivial structural motifs can be associated to the corresponding NBS length, we assigned to each match a weight proportional to this length.

3 The score function of the SSD_finder algorithm

In this section, we provided more details about the score function implemented in the core module *SSD_finder* of the MONSTER procedure.
Our SSD_finder is based on the score function

$$sc(C) = \sum_{i=1}^{n} P(m_i) + \sum_{i=1}^{n-1} Q(m_i, m_{i+1}) \tag{2}$$

that accounts for two different terms: P and Q. $P(m_i)$ is the weight of match m_i taking into account its individual relevance (i.e., the number of nucleotides). $Q(m_i, m_{i+1})$ is a weight taking into account how much the pair m_i, m_{i+1} in T has positions consistent with the corresponding NBSs

in R. In greater details, according to the definitions of "Material and Methods" section, we defined the $Q(m_i, m_{i+1})$ term as sum of two different terms: Q_1 and Q_2, if and only if such a difference is greater than zero, otherwise the $Q(m_i, m_{i+1})$ term is set to $-\infty$. Note that the score may evaluate $-\infty$, when $Q = -\infty$. This implies the rejection of chains containing matches whose positions on T are too different from the corresponding NBSs on R. In this way, a chain is automatically rejected. In particular,

$$Q_1(m_i, m_{i+1}) = GAP_{nbs} - [ind(nbs(m_{i+1})) - ind(nbs(m_i))] \tag{3}$$

and

$$Q_2(m_i, m_{i+1}) = GAP_{pos} - \frac{[pos(nbs(m_{i+1})) - pos(nbs(m_i))] - [pos(m_{i+1}) - pos(m_i))]}{[pos(nbs(m_{i+1}) - pos(nbs(m_i))]} \tag{4}$$

GAP_nbs and GAP_pos are two thresholds. The first one corresponds to the distance between $nbs(m_i)$ and $nbs(m_{i+1})$ beyond which Q_1 becomes negative. The second one corresponds to the discrepancy between the distances of reference and target NBSs. We choose $GAP_{nbs} = 3$ meaning that the $nbs(m_i)$ and $nbs(m_{i+1})$ are considered close if the distance between the corresponding NBSs in the reference is lower than 3; and the $GAP_{pos} = 0.1$ meaning that the distance between m_i and m_{i+1} in the target is considerable acceptable if the difference with the corresponding distance in the reference is at most 10%. Figure S1, additional file 4, shows an example of the relevance of the Q term: given a reference RNA sequence composed of four RSSPs and a target RNA sequence having six matches to them, two chains of matches can be extracted (chain 1 and chain 2 in the Figure S1, additional file 4). A shorter chain made of two RSSPs and a longer one made of four RSSPs. However, while the RSSP1-RSSP2 distance in the shorter chain is preserved, the same is distorted in the longer one. Thus, rewarding only the number of matched RSSPs (term P), the second chain would get the best score, neglecting the potentially most representative first chain.

4 SSD_finder validation procedure

In this section, we provided more details about the validation of our procedure, specifically designed to test the robustness of our SSD_finder algorithm.
Firstly, we tested the specificity of our SSD_finder evaluating its performances in the identification of members of four families obtained from the RFAM 11.0 database (http://rfam.sanger.ac.uk/). We chose the following representative RFAM families: (i) the Citrus tristeza virus replication signal (RFAM Acc.: RF00193), a regulatory element which plays a crucial role in the virus replication through its structures; (ii) the small ncRNAs OxyS family (RFAM Acc.: RF00035), induced in response to oxidative stress in Escherichia Coli; (iii) the lncRNAs family HAR1A (RFAM Acc.: RF00635), overlapping the Human Accelerated Region 1 (HAR1); and (iv) the lncRNAs family HOTAIRM1 (RFAM Acc.: RF01975), acting in myeloid transcriptional regulation). We applied MONSTER to each of the aforementioned families, the experimental workflow is the following: (i) the multiple sequences alignment of the family is used as the reference; (ii) a database of RNA sequences, including the four selected families and a subset of families randomly extracted from the RFAM and RNAstrand databases (more than 700 sequences in total), is used as the target; (iii) through RNAalifold, we obtain the consensus structure prediction of the family, that we use as input to nbRSSP_extractor to obtain the SSD of the reference; (iv) through Structator, we search for this reference SSD in the target; (v) the returned matches are used as an input to SSD_finder which computes the chains of matches with the highest score; note that, since the chains with

length one can be conceivably considered not significant, we filtered out them in this step. The chains returned by the MONSTER module SSD_finder for each family have been sorted in decreasing order with respect to the score in order to evaluate if this score can be able to discriminate the reference RFAM family among a width of false elements. We observed as our SSD_finder shows good performance for two families (RF00193 and RF00635), which are characterized by a quite specific SSD, consisting of 11 RSSPs and 4 complex RSSPs, respectively. For what concerns the other two families (RF00035 and RF01975), it is able to detect more than 80% of the members of families and achieves a high specificity, since it attained this result with a significantly low number of False positive values. Figure S1, additional file 4, shows the trend of the score computed by SSD_finder with respect to the target sequences to be covered. In every case, the score computed by SSD_finder drastically decreases approaching to the number of sequences that corresponds to the number of the family members, allowing a clear identification of the exact number of detected members. Thus, without a priori-knowledge about the True Positive values, the rapid decrease observed in the score of SSD_finder can be used as selection criterion of the sequences belonging to a given family. In fact, once the list of target sequences has been sorted based on the score, the number of elements belonging to a given reference family can be chosen as the value at which the jump occurs.

Moreover, we tested the SSD_finder performances in the identification of a whole sequence randomly chosen from those belonging to the RFAM families. In fact the RFAM families are composed of manually-curated fragment of sequences, while we aim to identify the whole sequence of such a fragment as belonging to the right RFAM family. We made up a target dataset composed of several RFAM families (i.e., more than 700 sequences belong to different families); (ii) we randomly chose a fragment of sequence that belongs to an RFAM family; (iii) we put at the head of the dataset the corresponding RFAM family to which the fragment selected belongs; and finally (iv) we used the corresponding whole sequence of the randomly chosen fragment as reference RNA. In that way, we added "noise" to our dataset, including several families of RNA sequence fragments as target dataset. We run our procedure, we ranked the results according to the decreasing order of the found scores and we found within the first three positions the right RFAM family to which belongs our selected sequence. Thus, we can conclude that our SSD_finder succeeds in finding the right sequence even if the target dataset is only composed of sequence segment, that made the detection harder. The ranking results are reported in Figure S3, additional file 6. The first column represent the sequences used as target (1=fragment of sequence belonging to the right detected Rfam family; 0=other sequences that do not belong to the detected Rfam family).
