# Supplementary material for: MONSTER v1.1: a tool to extract and search for RNA non-branching structures
Source: BMC Genomics. 2015 Jun 1;16(Suppl 6):S1. doi: 10.1186/1471-2164-16-S6-S1 (PMC4460781; doi:10.1186/1471-2164-16-S6-S1)
Supplement: Additional file 3 — It contains a detailed guide for MONSTER setting up and additional advanced information about the package algorithms and their application. [file 1471-2164-16-S6-S1-S3.pdf]

MONSTER v1.0

User’s Guide

Contents

Chapter 1: Getting Started **3**
MONSTER v 1.0 3
Pipeline overview 3
System requirements 4
Setting up 4
Additional files 5
Chapter 2: Basic Usage **6**
Aim of the tutorial 6
Preliminary steps 6
Preliminary files 6
Tutorial steps 6
Chapter 3: Advanced Information **13**
Non-branching extraction (nbRSSP_extractor) 13
Filtering (match_filter) 14
Chaining (SSD_finder) 15
Comparing NBSs (SSD_compare) 16
Chapter 4: Appendix **17**
File format specifications 17
Running the script MONSTER.sh 18

Getting Started 1

MONSTER_v1.0

MONSTER is a procedure to extract and search for RNA non-branching structures in order to identify common structural motifs.

Pipeline overview

The pipeline is composed of three parts (Figure UG1):

1. Structure prediction and encoding of the reference into secondary structural descriptor (SSD).
   a) prediction of the reference structure through *RNALfold*;
   b) extraction of the non-overlapping non-branching structures (NBSs) thorough *nbRSSP_extractor* module*;*
   c) encoding of SSD through *nbRSSP_extractor* module.

2. Matches searching and filtering.
   a) searching for matches between reference SSD and target sequence through *Structator*;
   b) filtering out of matches through *match_ filter* module.

3. Chains of matches building.
   a) building of chains of matches through *SSD_finder* module.

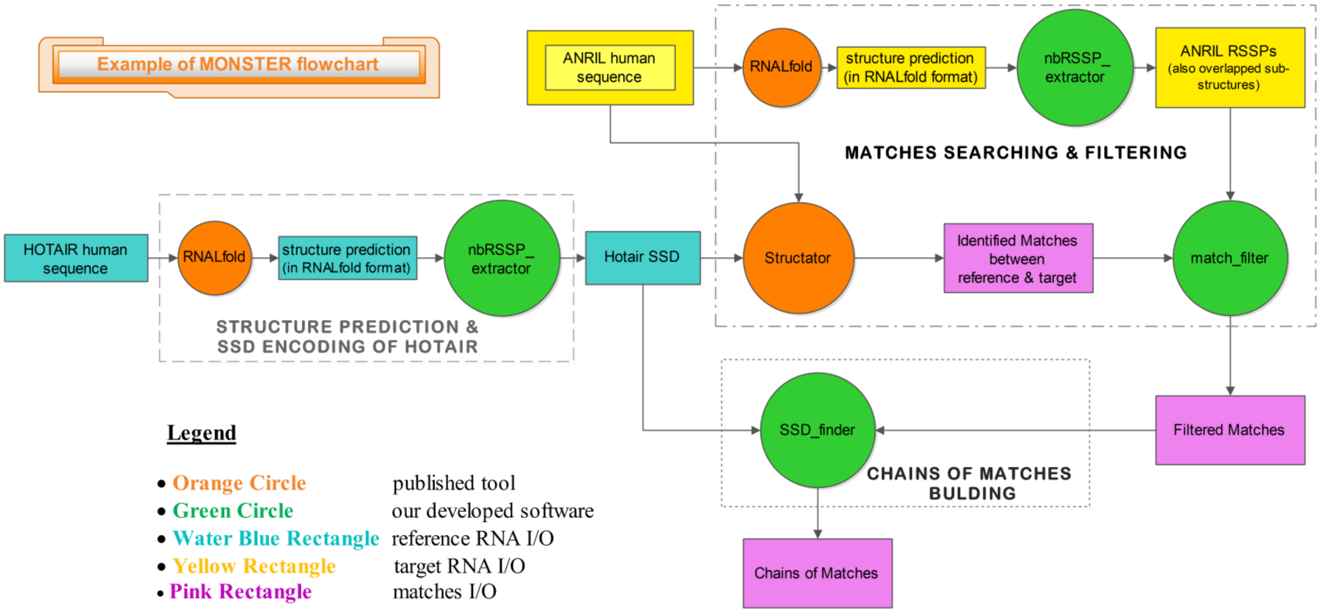

Figure UG1 Pipeline overview The pipeline is composed of three parts: (1) Structure prediction and SSD encoding of the reference (step 1-4 in the manuscript) (2) Matches searching and filtering (step 5-6 in the manuscript); (3) Chains of matches building (step 7 in the manuscript). More details are given in the paper. Such a flowchart is specific for the case in point of two RNA sequences (HOTAIR and ANRIL), explained in the tutorial of chapter 2.
Legend: orange circles represent published available tools; green circles represent software developed by us; rectangles represent software input and output (I/O), colored with water blue and yellow for what concerns reference and target, respectively.

System requirements

MONSTER version 1.1 has been tested on the following operative systems:

- Mac OS
- GNU/Linux (Ubuntu 12.04 or later; OS type: 64bit)
- Windows 7[1]

Setting up[2]

Download all necessary packages listed here:

- archive.zip file with MONSTER_v1.0 from the supplementary material of the paper;
- Structator1.1-linux-gnu.amd64.tar.gz (for Linux 64 bit) or select the best appropriate file for the user's operative system (http://www.zbh.uni-hamburg.de/en/research/application-oriented-bioinformatics/software/structator.html);
- ViennaRNA Package (http://www.tbi.univie.ac.at/~ronny/RNA/index.html). User can download the latest version of ViennaRNA Package selecting the own Package format according to his operative system. Otherwise older versions can be downloadable[3].

When all packages are downloaded:

- Install MONSTER_v1.0[4]
  - choose a directory and let "rootdir" be the path to this directory;
  - unzip the archive.zip file in "rootdir", that will create a folder named "archive";
  - type:

```
rootdir/archive> cd MONSTER_v1.0

rootdir/archive/MONSTER_v1.0> ls
```

  - you should find the directories:

```
bin      include     libs   src
```

  - type:

```
rootdir/archive/MONSTER_v1.0> cd src

rootdir/archive/MONSTER_v1.0/src> make
```

[1] To run MONSTER, some pre-existing packages are required (listed in the "Setting up" session). Some of these packages are not available for Window (e.g., *afsearch*). Thus, it is necessary to compile these packages on Window before running MONSTER.

[2] The attached distribution does not contain configuration files for Windows platform. A distribution including the configuration files for CMake is available on request. It can be used to generate the configuration files for Visual Studio.

[3] Source code of the latest version or older versions of the ViennaRNA package need to be compiled according to the guide lines provided in http://www.tbi.univie.ac.at/RNA/INSTALL.html.

[4] The instruction of MONSTER installation on GNU/Linux and Mac OS are even included in the "INSTALL" file of "archive/MONSTER_v1.0/src" folder.

- when the build process terminates you should find in the directory "rootdir/archive/MONSTER_v1.0/bin" the executables:

  `SSD_evaluator    SSD_finder  match_filter   nbRSSP_extractor`

- Install Structator1.1
  - uncompress Structator1.1-linux-gnu.amd64.tar.gz;
  - go the subfolder "bin" of the unzipped "Structator1.1-linux-gnu.amd64" file;
  - copy the executable *afsearch* [5] according to the user operative system in "MONSTER v1.0/bin".

- Install ViennaRNA Package
  - copy the executable *RNALfold* [6] according to the user operative system (e.g., *RNALfold.exe* on Window platform) in "MONSTER v1.0/bin". On unix platform (e.g., Mac OS, GNU/Linux) you can find the executable path using the command: `which RNALfold` (e.g., /usr/bin RNALfold).

Finally, in the directory "archive/MONSTER/bin" you should find the following executable files:

- afsearch
- RNALfold
- nbRSSP_extractor
- match_filter
- SSD_evaluator
- SSD_finder

To test if the executables have been correctly built type, run:

```
1) ./afsearch
2) ./RNALfold -h
3) ./nbRSSP_extractor -h
4) ./match_filter -h
5) ./SSD_evaluator -h
6) ./SSD_finder -h
```

The online help should be displayed for each command.

Additional files:

The "data" subfolder of "archive" contains the following additional files, needed to run MONSTER:

- dna_rna.comp[7];
- rna.alphab[8].

[5] *afSearch* is a program for matching RNA sequence-structure patterns in a precomputed index or directly in a plain FASTA file.
[6] *RNALfold* is a program for calculating locally stable secondary structures of RNAs.
[7] File specifying the Watson-Crick and wobble complementary rules.
[8] File specifying an alphabet to which characters are mapped and the sequences are then alphabetically transformed, needed to run *afsearch* program (see the user's manual v1.01 of Structator packages for details).

Basic Usage 2

After installing MONSTER software, you can follow a sample run executing the following tutorial[9].

Aim of the tutorial:

The user whishes to search for chains (group of matches) of a reference lncRNA into a target lncRNA. For this example we considered as a reference HOTAIR and as a target ANRIL.

Preliminary step:

Go to the "archive/example_data" subfolder which stores all the file needed to execute the tutorial. You can run the tutorial step by step following the "tutorial step" section. Otherwise, you can run the script "MONSTER.sh" on unix platforms to execute the whole tutorial procedure.

Preliminary files:

- HOTAIR_human.fasta: a fasta file with the RNA sequence of HOTAIR
- ANRIL_human.fasta: a fasta file with the RNA sequence of ANRIL

Tutorial Steps:

1
```
../MONSTER_v1.0/bin/RNALfold  <HOTAIR_human.fasta -L 150 \
>HOTAIR_human_RNALfold_150_pred.txt
```

2
```
../MONSTER_v1.0/bin/nbRSSP_extractor  -i HOTAIR_human_RNALfold_150_pred.txt  \
-o  HOTAIR_human_RNALfold_150_pred_ssd.pat
```

3
```
../MONSTER_v1.0/bin/afsearch ANRIL_human.fasta -comp ../data/dna_rna.comp -alph ../data/rna.alphab  \
-pat   HOTAIR_human_RNALfold_150_pred_ssd.pat -t HOTAIRvsANRIL_matches.txt
```

4
```
../MONSTER_v1.0/bin/RNALfold <ANRIL_human.fasta -L 150
>ANRIL_human_RNALfold_150_pred.txt
```

5
```
../MONSTER_v1.0/bin/nbRSSP_extractor -i  ANRIL_human_RNALfold_150_pred.txt \
-o   ANRIL_human_RNALfold_150_pred_ssd_com.txt --com --RNALfold_out
```

6
```
../MONSTER_v1.0/bin/match_filter -r  ANRIL_human_RNALfold_150_pred_ssd_com.txt  \
-m  HOTAIRvsANRIL_matches.txt -o HOTAIRvsANRIL_filtered.txt --com
```

7
```
../MONSTER_v1.0/bin/SSD_finder -s HOTAIR_human_RNALfold_150_pred_ssd.pat  \
-m  HOTAIRvsANRIL_filtered.txt -o HOTAIR_chains.txt --com
```

[9] We provide the command lines to run the tutorial on unix platforms (i.e., GNU/Linux, Mac OS).

Details of each step are explained as follows:

1. Run *RNALfold* of the *Vienna Package* to obtain the secondary structure predictions for the HOTAIR sequence in dot-bracket notation [10].

   Synopsis:

   RNALfold.exe [-L span]

   Description:

   RNALfold reads RNA sequences from stdin and prints local structure predictions to stdout.

   Options:

   -L span
   Set the maximum allowed separation of a base pair to span, i.e. no pairs (i,j) with j-i>L will be allowed. In the present example, we used L = 150.

   Command line example:

```
../MONSTER_v1.0/bin/RNALfold <HOTAIR_human.fasta -L 150 \
>HOTAIR_human_RNALfold_150_pred.txt
```

   Input file: "HOTAIR_human.fasta".
   Output file: "HOTAIR_human_RNALfold_150_pred.txt".

   The format of the output file is as follows:

```
>hotair_human
```
Sequence header

Local predicted structure (dot-bracket notation)

Free energy (kcal/mol)

Starting position in the sequence

```
.((((((.....)))))). ( -2.60) 2317
.(((((......))))).((((((.....)))))). ( -3.10) 2300
.(((((((((.....))))))))).......((((((.....)))))). ( -8.20) 2287

                                        [...]

.((((((...)))))) ( -4.10)   15
.(((((..(..((((((...))))))..)..))))) (-14.70)    5
```

Nucleotides sequence

```
ACAUUCUGCCCUGAUUUCCGGAACCUGGAAGCCUAGGCAGGCAGUGGGGAACUCUGACUCGCCUGUGCUCUGGAGCUUGAUCCGAAAG
CUUCCACAGUGAGGACUGCUCCGUGGGGGUAAGAGAGCACCAGGCACUGAGGCCUGGGAGUUCCACAGACCAACACCCCUGCUCCUGG

                                        [...]

UGGUUUUAUAUGCCUUAUGGAGUAUAUACUCACAUGUAGCUAAAUAGACUCAGGACUGCACAUUCCUUGUGUAGGUUGUGUGUGUGUG
GUGGUUUUAUGCAUAAAUAAAGUUUUACAUGUGGUGAAAAAA
 (-691.04)
```
Minimum free energy

[10] dots represent unpaired nucleotides; matched brackets (opened/closed) represent paired nucleotides.

2. Run *nbRSSP_extractor* to extract the NBSs using the “HOTAIR_human_RNALfold_150_pred.txt” file as input. The software returns the HOTAIR SSD comprising of 67 RSSPs.

Command line example:

```
../MONSTER_v1.0/bin/nbRSSP_extractor -i HOTAIR_human_RNALfold_150_pred.txt \
-o HOTAIR_human_RNALfold_150_pred_ssd.pat
```

Input file: “HOTAIR_human_RNALfold_150_pred.txt”.
Output file: “HOTAIR_human_RNALfold_150_pred_ssd.pat”.

The format of the output file is as follows:

```
>RSSP0|startpos=6|occurrences=1|weight=3.50|cumulative_fe=0.408333|   ] NBS details
len=35|loop_pos=(21,25)|seq_len=2337                                  ]
NNNNNNNNNNNNNNNNNNNNNNNNNNNNNNNNNNN  <— NBS sequence (wild-characters)
(((((..(..((((((...))))))..)..)))))  <— NBS

>RSSP1|startpos=42|occurrences=1|weight=1.50|cumulative_fe=0.423944|
len=15|loop_pos=(47,52)|seq_len=2337
NNNNNNNNNNNNNNN
(((.((....)))))

        [...]

>RSSP65|startpos=2300|occurrences=5|weight=1.80|cumulative_fe=1.413313|
len=18|loop_pos=(2305,2312)|seq_len=2337
NNNNNNNNNNNNNNNNNN
((((((......))))))

>RSSP66|startpos=2318|occurrences=7|weight=1.70|cumulative_fe=1.359844|
len=17|loop_pos=(2323,2329)|seq_len=2337
NNNNNNNNNNNNNNNNN
((((((.....))))))
```

3. Run the *afSearch* program of *Structator* package to look for reference SSD of HOTAIR into the target ANRIL sequence, setting a global modality. The software returns the found matches.

Command line example:

```
../MONSTER_v1.0/bin/afsearch ANRIL_human.fasta -comp ../data/dna_rna.comp \
-alph ../data/rna.alphab -pat HOTAIR_human_RNALfold_150_pred_ssd.pat -t
HOTAIRvsANRIL_matches.txt
```

Input files: “ANRIL_human.fasta” (target), “HOTAIR_human_RNALfold_150_pred_ssd.pat” (reference), “dna_rna.comp”, and “rna.alphab”.

Output file: “HOTAIRvsANRIL_matches.txt”.

The *Structator* output is in the following format:

```
![matched substring][structure][seq. id][matching pos.][pattern id][weight][strand]

       GCUACAU        (.....)        0      1      39     100    f
       GCUACAUCCGU    ((.......))    0      1      15     110    f
       GCUACAUCCGU    ((.......))    0      1      54     110    f
       ACAUCCGU       ((....))       0      4      7      100    f
       ACAUCCGU       ((....))       0      4      11     100    f
       ACAUCCGU       ((....))       0      4      16     100    f
       ACAUCCGU       ((....))       0      4      17     100    f
       ACAUCCGU       ((....))       0      4      34     100    f
       ACAUCCGU       ((....))       0      4      48     100    f
       CAUCCG         (....)         0      5      9      100    f

                                      [...]

       UGGGCUCA       ((....))       0      3818   48     100    f
       GGGCUC         (....)         0      3819   9      100    f
       GGGCUC         (....)         0      3819   22     100    f
       GGGCUC         (....)         0      3819   40     100    f
       UCAGACA        (.....)        0      3823   39     100    f
       AGACAAU        (.....)        0      3825   39     100    f
       GACAAU         (....)         0      3826   9      100    f
       GACAAU         (....)         0      3826   22     100    f
       GACAAU         (....)         0      3826   40     100    f
       UAAAAA         (....)         0      3831   9      100    f
       UAAAAA         (....)         0      3831   22     100    f
       UAAAAA         (....)         0      3831   40     100    f
       UAAAAAA        (.....)        0      3831   39     100    f
```

4. Run *RNALfold* (with a span L equal to 150) to obtain the secondary structure predictions of the ANRIL sequence.

Command line example:

```
../MONSTER_v1.0/bin/RNALfold <ANRIL_human.fasta -L 150 \
>ANRIL_human_RNALfold_150_pred.txt
```

Input file: “ANRIL_human.fasta”.
Output file: “ANRIL_human_RNALfold_150_pred.txt”.

The output format is the same of step 1.

5. Run *nbRSSP_extractor* to extract the non-branching structures (NBSs) from the “ANRIL_human_RNALfold_150_pred.txt” file, retaining even the overlapped RSSPs. Thus, we have a wide array of possible structure predictions of the target.

   Command line example:

```
../MONSTER_v1.0/bin/nbRSSP_extractor -i ANRIL_human_RNALfold_150_pred.txt \
-o ANRIL_human_RNALfold_150_pred_ssd_com.txt --com --RNALfold_out
```

   Input file: “ANRIL_human_RNALfold_150_pred.txt”.
   Output file: “ANRIL_human_RNALfold_150_pred_ssd_com.txt”.

   The output format is the same of step 2, the only difference consists of the higher number of RSSPs that are extracted, because the option --*RNAlfold_out* allows to maintain even overlapped predictions.

6. Run *match_filter* to discard the unlikely matches obtained running the step 5, based on the predicted RSSPs of step 5. The software returns the filtered matches between HOTAIR and ANRIL.

   Command line example:

```
../MONSTER_v1.0/bin/match_filter -r ANRIL_human_RNALfold_150_pred_ssd_com.txt \
-m HOTAIRvsANRIL_matches.txt -o HOTAIRvsANRIL_filtered.txt --com
```

   Input files: “ANRIL_human_RNALfold_150_pred_ssd_com.txt”, “HOTAIRvsANRIL_matches.txt”.
   Output file: “HOTAIRvsANRIL_filtered.txt”.

   The output format is the same of the step 3, but with a lower number of matches because of the filtering.

7. Run *SSD_finder* to perform the chaining. It returns the chains of matches that represent the structural motif shared between ANRIL and HOTAIR.

   Command line example:

```
../MONSTER_v1.0/bin/SSD_finder -s HOTAIR_human_RNALfold_150_pred_ssd.pat \
-m HOTAIRvsANRIL_filtered.txt -o HOTAIR_chains.txt --com
```

   Input files: “HOTAIR_human_RNALfold_150_pred_ssd.pat” (reference),
   HOTAIRvsANRIL_filtered.txt” (matches).

   Output files: “HOTAIR_chains.txt”.

The format of the output file is as follows:

```
# <seqID = 0>    <-- Target sequence identifier

Chain score   Pattern ID (reference)   Weight   Position in the target sequence

Chain [ score 3.99  | pID=30, w=1.20, pos=41 | pID=32, w=1.00, pos=146 | pID=34, w=1.00,
      [ pos=207 | dist=(116,105) | dist=(64,61)   <-- Relative Distances between pID in (reference, target)

score 2.80  | pID=21, w=1.00, pos=182 | pID=22, w=1.00, pos=208 | dist=(25,26)

score 2.56  | pID=30, w=1.20, pos=205 | pID=32, w=1.00, pos=312 | dist=(116,107)

score 2.73  | pID=30, w=1.20, pos=205 | pID=32, w=1.00, pos=326 | dist=(116,121)

                              [...]

score 4.39  | pID=7, w=1.00, pos=2755 | pID=11, w=1.00, pos=2827 | pID=14, w=1.00,
pos=2960 | pID=17, w=1.00, pos=3045 | dist=(75,72) | dist=(141,133) | dist=(79,85)

score 4.11  | pID=11, w=1.00, pos=3625 | pID=14, w=1.00, pos=3767 | pID=15, w=1.10,
pos=3787 | dist=(141,142) | dist=(22,20)

score 2.75  | pID=9, w=1.00, pos=3769 | pID=11, w=1.00, pos=3819 | dist=(50,50)

score 3.98  | pID=30, w=1.20, pos=3654 | pID=32, w=1.00, pos=3757 | pID=34, w=1.00,
pos=3819 | dist=(116,103) | dist=(64,62)
```

The first line (starting with "#") contains the number of target sequence (in this case <seqID = 0> because ANRIL is the only target sequence analyzed); then, there is a line for each found chain of matches. Each line starts with the computed score of the chain, and it is followed by (i) the pattern ID (*pID*) of the reference RSSPs found in the target sequence; (ii) the positions (*pos*) at which RSSPs have been found in the target; (iii) the weight (*w*) of each RSSP; and (iv) the pair-wise relative distances (*dist*). This parameter consists of two numbers enclosed in the brackets and comma-separated: the first providing the distance of the found RSSPs in the reference and the second representing the corresponding distance in the target. The highest scores represent the most putative structural motifs shared between the reference and the target.

Advanced Information 3

This chapter explains the details of our implemented algorithms.

Non-branching structure Extractor (nbRSSP_extractor)

nbRSSP_extractor generates a file containing the list of non-branching RSSPs (SSD) extracted from the input. The input file is a list of sequence/description-of-structure pairs that may be provided in several formats; one SSD for each pair is generated; non-branching RSSPs may produced according to different algorithms. The default input format is the output of RNALfold. Since in this case many overlapping substructures are provided for each sequence two different strategies may be used to select the RSSPs forming the SSD: (i) “linearization” (option -RNALfold_lnrz) meaning that non-overlapping substructures are first selected according to increasing free energy and then are extracted the RSSPs, (ii) “weighted” (default) meaning that all possible RSSPs are extracted first from overlapping substructures and weighted with the absolute value of the “mean free energy” (i.e., a free energy per nucleotide normalized with the structure length) and then the non-overlapping extracted RSSPs are selected according to decreasing weight; in the weighted case, with the option -RNALfold_out, all the RSSPs extracted from overlapping substructures are directly sent to output without any selection.

1. **Synopsis**

   nbRSSP_extractor.exe [-f <string>] [-i <string>] [-o <string>] [--com] [--fmtOut <string>] [--sort <string>] [-s] [--RNALfold_lnrz] [--RNALfold_out] [--] [--version] [-h]

2. **Input**
   - RNALfold (default): output of RNALfold
   - Rfold: output of Rfold (only one sequence/description-of-structure pair at the time), or
   - Sfold: output of Sfold (only one sequence/description-of-structure pair at the time), or
   - seq-struct: output of RNAfold, or
   - seqs-structs: general sequence/structure pairs possibly preceded by a FASTA header.

3. **Output**
   List of non-branching RSSP (SSD) extracted from the predicted structures

4. **Options**
   - -f <string>, --fmtIn <string>

     Input file format [RNALfold|Rfold|Sfold|seq-struct|seqs-structs] (default: RNALfold)

   - -i <string>,--fin <string>

     Input file name

   - -o <string>, --fout <string>

     Output file name

   - --com

Enable insertion of comments in the output

- --fmtOut <string>

  Output file format [structator|fasta] (default: structator)

- --sort <string>

  Sort criterium for RSSP selection [sort_by_nfe|sort_by_cfe|sort_by_occs] (default: sort_by_nfe)

- -s, --seq

  Print sequence in the RSSP descriptors (default: prints N)

- --RNALfold_lnrz

  Enable RSSP extraction from a linearization of input produced by RNALfold

- --RNALfold_out

  Generate a file containing descriptors of all RSSP predicted by RNALfold (default: the file is not generated)

- --, --ignore_rest

  Ignores the rest of the labeled arguments following this flag

- --version

  Displays version information and exits

- -h, --help

  Displays usage information and exits

Matches Filtering (match_filter)

Match_filter filters out matches that cannot actually fold. It writes a file of matches (following the output format of *Structator*) containing for each sequence the matches that have been someway predicted. Current implementation considers a match predicted if it is a substructure of some predicted RSSP. In particular the external loop of the match must coincide with the one of the predicted RSSP.

1. **Synopsis**

   match_filter.exe -r <string> [-m <string>] [-o <string>] [--com] [--] [--version] [-h]

2. **Input**
   - a list of matches corresponding to one or more sequences as generated by *Structator;*
   - a list of SSDs, one for each sequence present in the list of matches, corresponding to non-branching RSSP predicted by some prediction tool for those sequences.

3. **Output**
   List of filtered matches between reference and target.

4. **Options**

- -r <string>, --RSSP <string>

(required) Input file name of predicted non-branching RSSPs

- -m <string>, --match <string>

  Input file name of matches

- -o <string>, --fout <string>

  Output file name

- --com

  Enable insertion of comments in the output

- --, --ignore_rest

  Ignores the rest of the labeled arguments following this flag

- --version

  Displays version information and exits

- -h, --help

  Displays usage information and exits

Chaining (SSD_finder)

SSD_finder finds most significant local groups of matches in a target file that have correspondence in a given reference SSD.

1. **Synopsis**

   SSD_finder -s <string> [-m <string>] [-o <string>] [--com] [--] [--version] [-h]

2. **Input**

- Reference SSD to be searched for;
- List of matches founded between reference and target.

3. **Output**

   Chain of matches.

4. **Options**

- -s <string>, --SSD <string>

  (required) Input file name of the reference SSD

- -m <string>, --match <string>

  Input file name of matches

- -o <string>, --fout <string>

  Output file name

- --com

  Enable insertion of comments in the output

- --, --ignore_rest

  Ignores the rest of the labeled arguments following this flag.

- --version

  Displays version information and exits.

- -h, --help

  Displays usage information and exits.

Comparing non-branching structure option (SSD_compare)

SSD_compare generates statistics on the comparison between a list of reference SSDs and a corresponding list of target SSDs.

1. **Synopsis**

   SSD_compare.exe -r <string> [-t <string>] [-o <string>] [-v] [--] [--version] [-h]

2. **Input**
   - Reference SSD;
   - Target SSD.

3. **Output**
   - some statistics for each RSSP of an SSD (lines beginning with <r1> only when option -v, --verbose is set);
   - some statistics for each SSD (lines beginning with <r2>)
   - comment lines (lines beginning with the '#' character) contain information aimed to make the file readable.

4. **Options**
   - -r <string>, --ref <string>

     (required) Input file name of reference SSDs

   - -t <string>, --target <string>

     Input file name of target SSDs

- -o <string>, --fout <string>

  Output file name

- -v, --verbose

  Print complete SSD scores (default: print only global SSD scores)

- --, --ignore_rest

  Ignores the rest of the labeled arguments following this flag.

- --version

  Displays version information and exits.

- -h, --help

  Displays usage information and exits.

Appendix 4

File format specifications

The RNA sequence chosen as reference and target in the "Basic Usage" session are the human lncRNA HOTAIR (2354 nucleotides) and ANRIL (3858 nucleotides) in FASTA format, respectively.

The FASTA format requires a single description line, followed by lines of nucleotides sequence. The description line begins with a greater-than (">") symbol that separates the sequence identifiers from the sequence data. Black lines are not allowed in the middle of the file.

An example for the reference and target sequences in FASTA format is shown:

```
>HOTAIR_human
acattctgccctgatttccggaacctggaagcctaggcaggcagtggg
gaactctgactcgcctgtgctctggagcttgatccgaaagcttccaca
gtgaggactgctccgtgggggtaagagagcaccaggcactgaggcctg
ggagttccacagaccaacacccctgctcctggcggct.....
```

```
>ANRIL_human
agctacatccgtcacctgacacggccctaccaggaacagccgcgctc
ccgcggattctggtgctgctcgcgtccccgctcccctattcccctta
ttttattcctggctcccctcgtcgaaagtcttccattcttcaaacta
gattatttaaaaatgaaaaaggaagaaaggaaagcgag....
```

Both sequences are included in the subdirectory "example data" of the archive.zip file (called "HOTAIR_human.fasta" and "ANRIL_human.fasta"). However, they can be downloaded from the online database lncRNAdb (http://www.lncrna.org/) with identifiers Gm16258 and CDKN2B-AS1for HOTAIR and ANRIL lncRNAs, respectively.

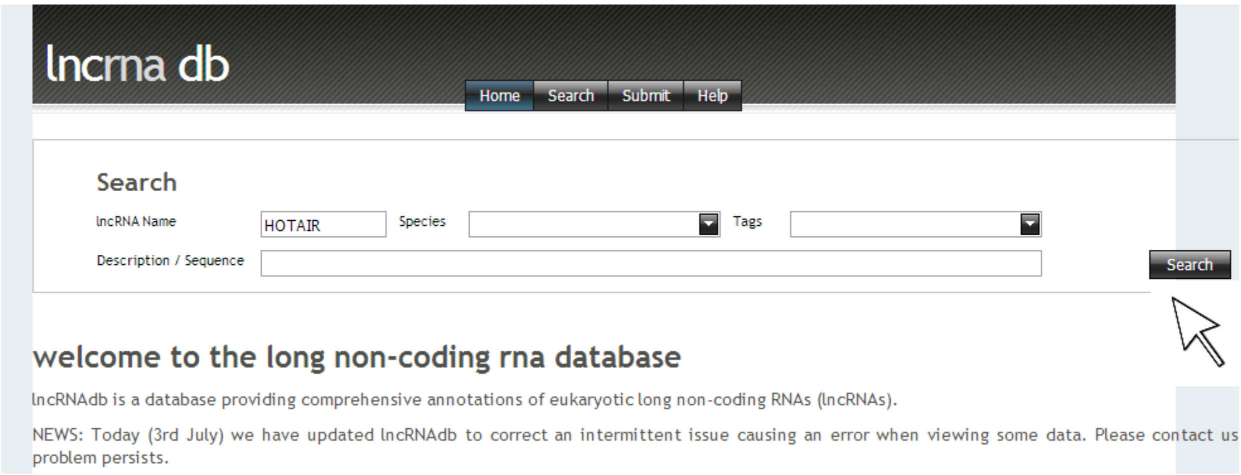

Running the Script MONSTER.sh

MONSTER.sh searches for chains (group of matches) of a reference RNA into a target RNA.

1. **Synopsys**

   MONSTER [-L span] [-h] <REFERENCE-FILE> <TARGET-FILE>

2. **Input file**
   - REFERENCE-FILE = File in fasta format of the reference RNA (e.g., HOTAIR_human.fasta)
   - TARGET-FILE = File in fasta format of the target RNA (e.g., ANRIL_human.fasta)

3. **Output file**
   Chains of the reference RNA into the target RNA

4. **Options**
   - - L:
     Set maximum base pair separation to “span” (default = 150);

   - -h
     Displays usage information and exits.

Command line example:

```
sh ./MONSTER.sh HOTAIR_human.fasta ANRIL_human.fasta
```
