# Supplementary figures and images for: MONSTER v1.1: a tool to extract and search for RNA non-branching structures
Source: BMC Genomics. 2015 Jun 1;16(Suppl 6):S1. doi: 10.1186/1471-2164-16-S6-S1 (PMC4460781; doi:10.1186/1471-2164-16-S6-S1)

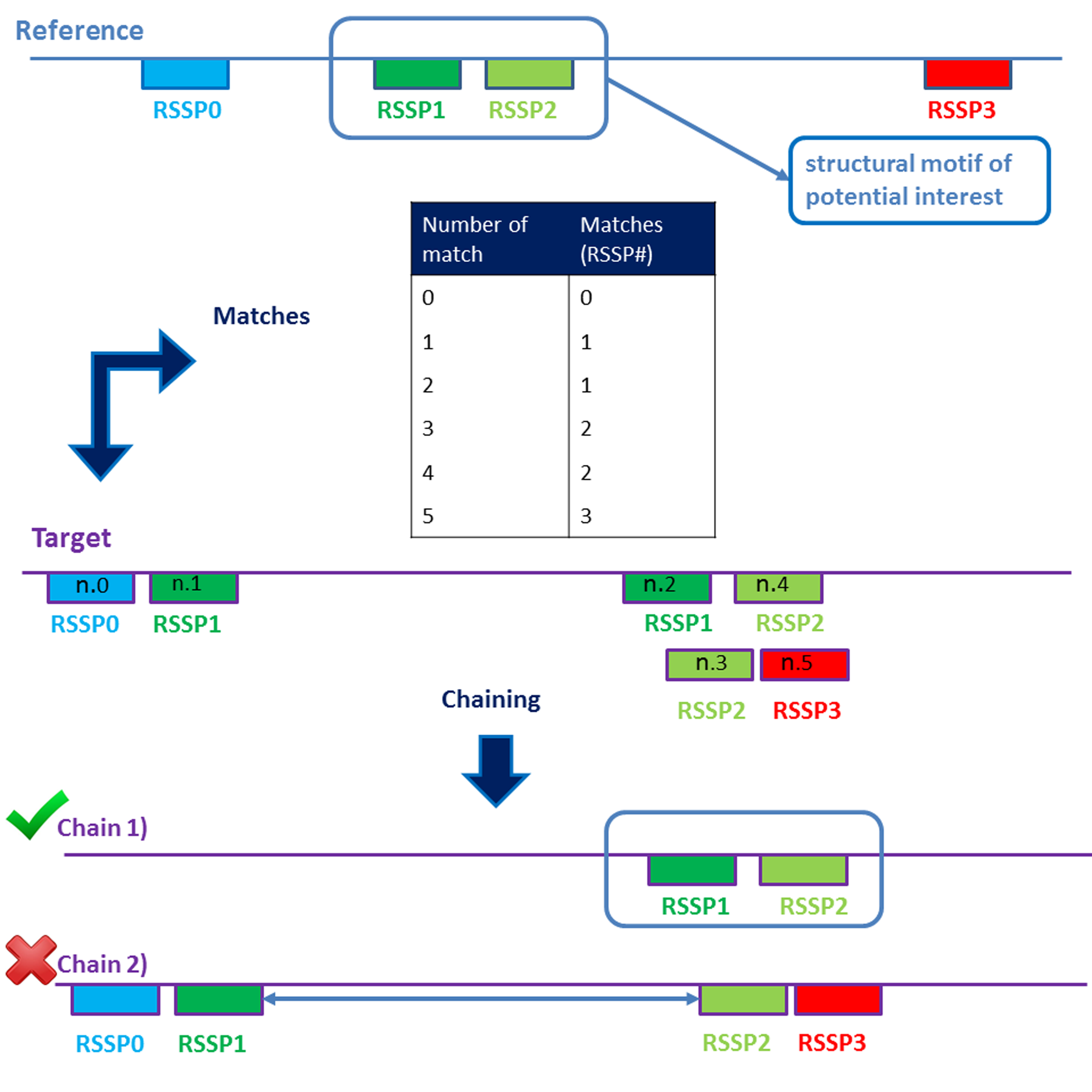

Supplement: Additional file 4 — Figure S1 - Relevance of the Q term of score function (1). It contains the supplementary figure that depicts an example of the Relevance of the Q term of the score function into chaining. An example of the chaining step (step 7) that shows the relevance of evaluating the distance among the RSSPs along with the number of RSSPs to select the best chain of matches. [file 1471-2164-16-S6-S1-S4.png]

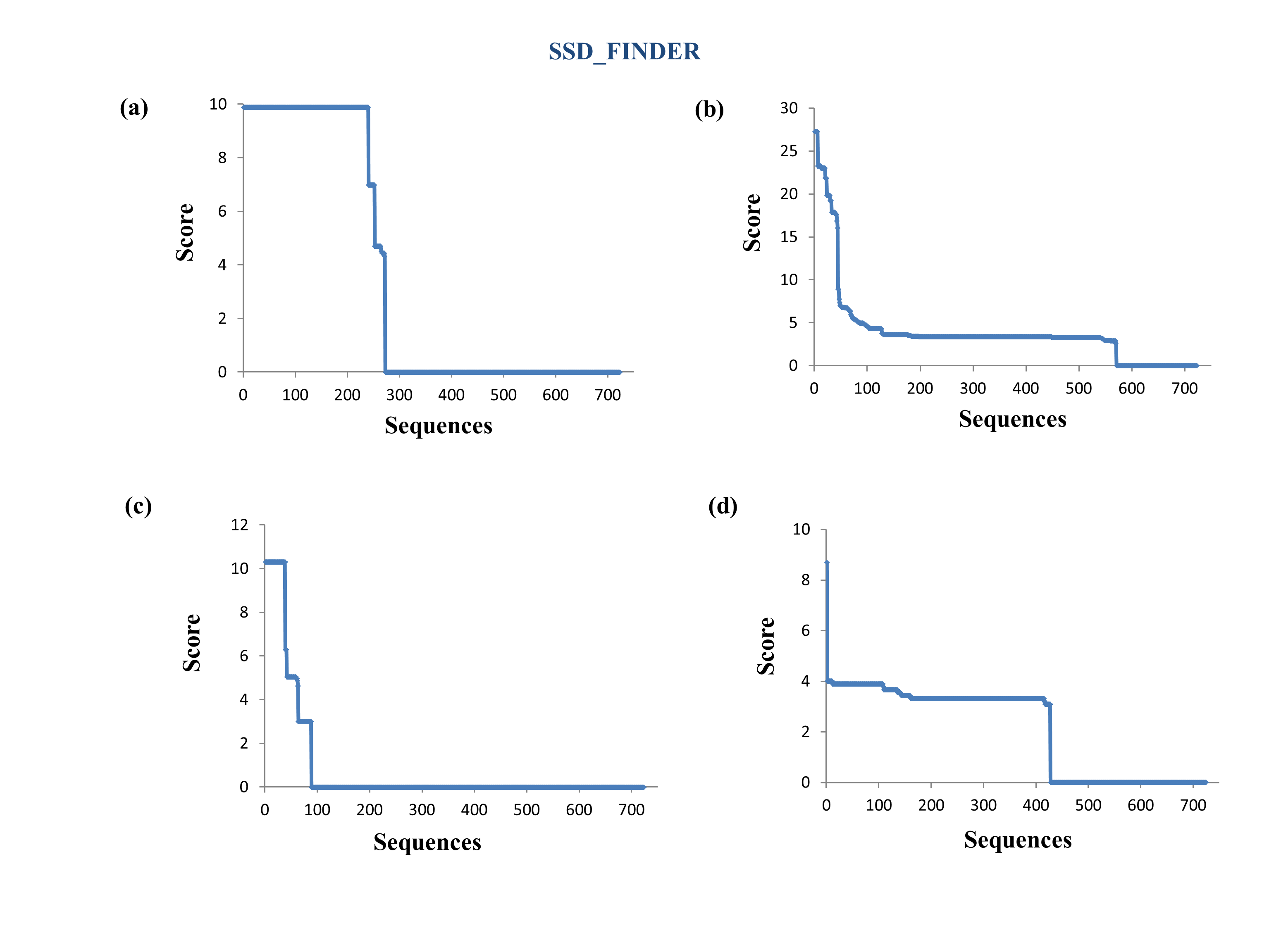

Supplement: Additional file 5 — Figure S2 - Chain scores evaluated from SSD_finder. It contains the supplementary figure that depicts the chain scores evaluated from SSD_finder. Each panel represents the efficiency of our SSD_finder in the classification of the members of the four analyzed Rfam families (i.e., (a) RF00193, (b) RF00035, (c) RF00635, and (d) RF1975). The y axes represent the score of our algorithm, evaluated as in equation (1) in our manuscript; the × axis represents the number of RNA sequences that constitute the database used as target in the chaining validation. This database includes the four selected families and a subset of families randomly extracted from the Rfam and RNAstrand databases (more than 700 sequences in total). In each case, the score computed by SSD_finder drastically decreases approaching to the number of sequences that corresponds to the number of the family members, allowing a clear identification of the exact number of detected members. [file 1471-2164-16-S6-S1-S5.png]

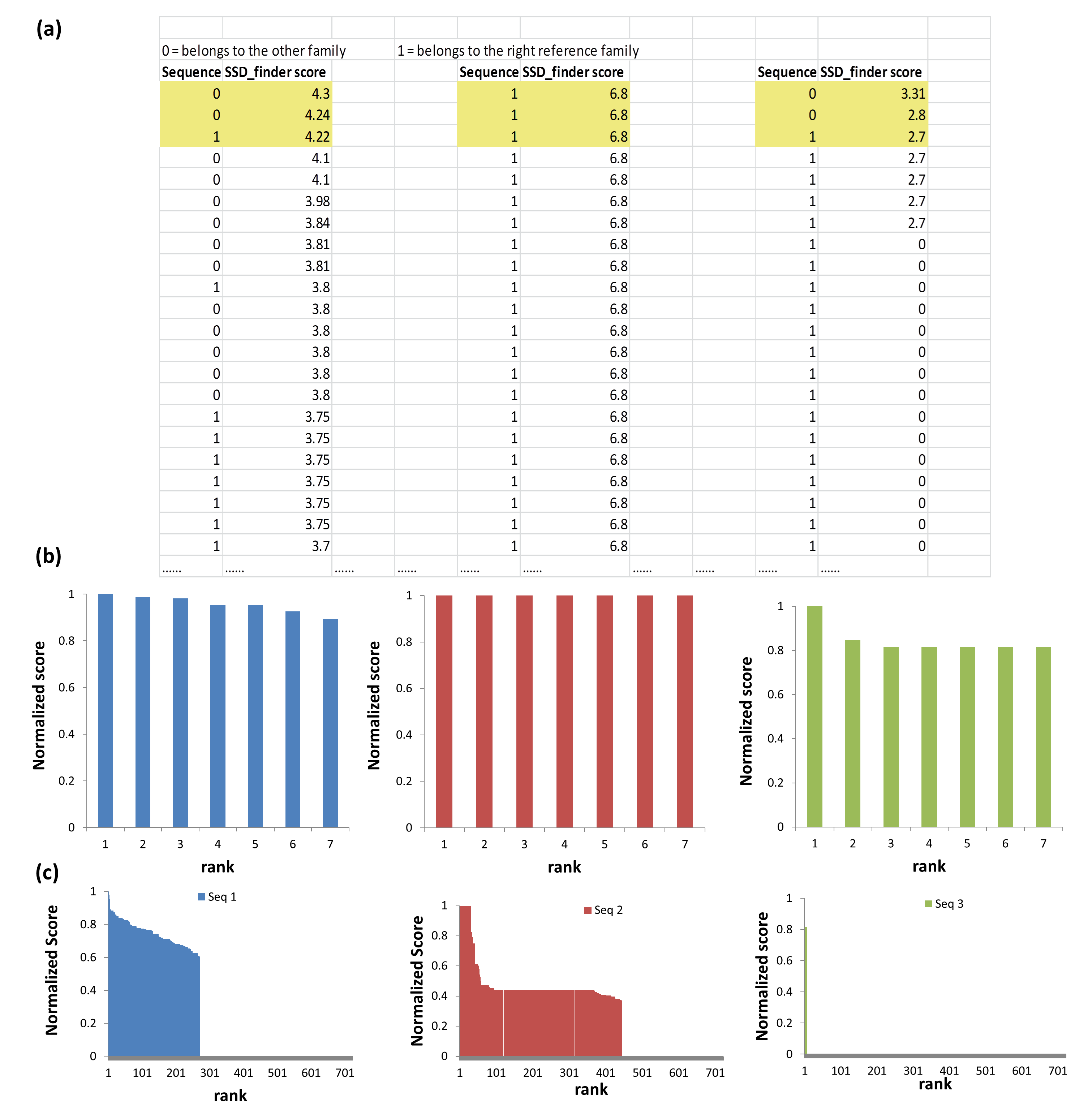

Supplement: Additional file 6 — Figure S3 - Ranking results of the chain score of SSD_finder in the validation procedure. It contains the supplementary figure that depicts the rank results. Each panel represents the efficiency of our SSD_finder in identifying the right Rfam family in at least the three positions. (a) Table with the ranking results, whose first column represents the sequences used as target (1=fragment of sequence belonging to the right detected Rfam family; 0=other sequences that do not belong to the detected Rfam family). (b), (c) Bar plots that represent the graphical representation of the ranking results. The y axes represent the normalized score of our algorithm, evaluated as in equation (1) in our manuscript; the × axes represent the number of RNA sequences that constitute the database used as target in the chaining validation. This database includes the Rfam family to which the whole sequence used as reference belongs and a subset of families randomly extracted from the Rfam database (more than 700 sequences in total). The first group of plots (b) sketches the first seven ranking results, the second one (c) represents the whole set of sequences. [file 1471-2164-16-S6-S1-S6.png]

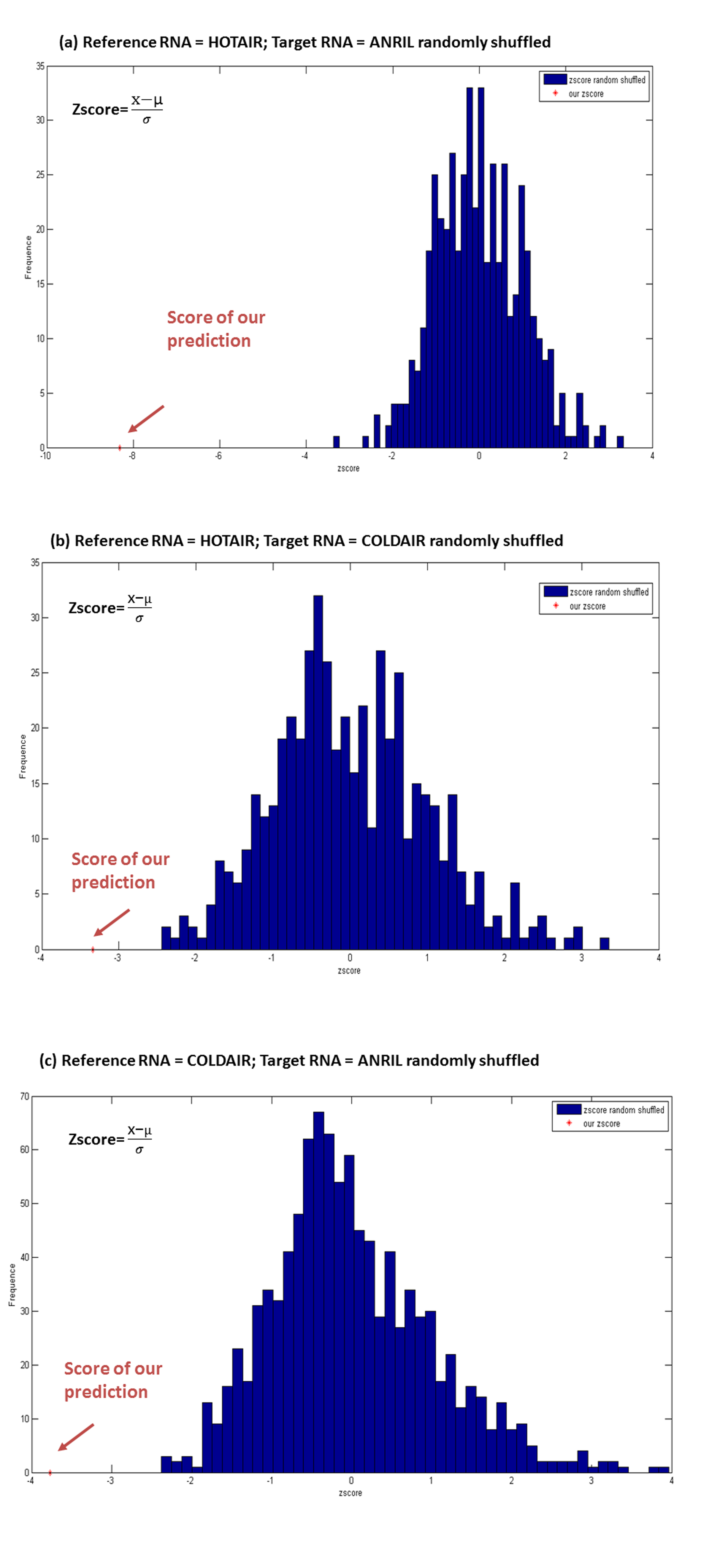

Supplement: Additional file 7 — Figure S4 - Distribution of the zscores. It contains the supplementary figure that depicts the zscore distributions. Each panel represents the score distribution of zscore using a shuffled version of the lincRNA joint with the score of our original comparison: (a) HOTAIR has been chosen as reference and the shuffled versions of ANRIL as target; (b) HOTAIR has been chosen as reference and the shuffled versions of COLDAIR as the target; (c) COLDAIR has been chosen as reference and the shuffled versions of ANRIL as target. It has been noticed how in all the cases our score is statistically significant with respect to the random distribution, yielding on the tails of distribution and corresponding to a p-value ≈0. [file 1471-2164-16-S6-S1-S7.png]
